# A critical review on temperature-mediated marine plastic biodegradation

**DOI:** 10.1016/j.eehl.2025.100177

**Published:** 2025-08-20

**Authors:** Yuanmei Zhang, Yiqi Cao, Bing Chen, Baiyu Zhang

**Affiliations:** Northern Region Persistent Organic Pollution Control (NRPOP) Laboratory, Faculty of Engineering and Applied Science, Memorial University, St. John's, NL A1B 3X5, Canada

**Keywords:** Plastics biodegradation, Marine environments, Temperature impacts, Plastisphere, Enzymatic depolymerization, Plastics degrading-bacteria

## Abstract

Biodegradation offers a promising solution to marine plastic pollution. Temperature plays a significant role in biofilm development and microbial dynamics. However, comprehensive studies on the effects of temperature on marine plastic biodegradation remain limited, as most research focuses on individual and moderate temperatures, overlooking how temperature variations across polar to tropical marine environments interact with other ecological factors to influence plastic biodegradation. This review summarizes current research on temperature-induced biofilm formation, microbial succession, and enzymatic depolymerization of plastics. The findings reveal that higher temperatures generally enhance biofilm growth. Notably, cold-tolerant bacteria stimulate the production of extracellular polymeric substances (EPS) to stabilize biofilms and adapt to cold conditions. Microbial succession, particularly within the Proteobacteria phylum, is primarily regulated by temperature, driving shifts in microbial diversity and activity. For different types of plastics, the hydrolyzable ones are degraded via enzymes such as cutinases, lipases, and depolymerases, mostly at mild temperatures. In contrast, non-hydrolyzable plastics are relatively recalcitrant to enzymatic breakdown but can be biodeteriorated by enzyme-generated reactive oxygen species (ROS), with minimal temperature influence due to their slow biodegradation. This review emphasizes the critical role of temperature in biodegradation processes and prospects for promising strategies for improving marine plastic management under the changing climate.

## Highlights

• Temperature impacts on marine plastic biodegradation across polar to tropical climates is explored.
• Lower temperature enhances biofilm stability through increased EPS production, while higher temperature accelerates early biofilm formation.
• Proteobacteria is reported as the dominant phylum for marine plastic degradation within moderate temperatures (15 ​°C-30 ​°C).
• Distinct temperature-dependent degradation behaviors of hydrolyzable plastics (e.g., PHAs) and non-hydrolyzable plastics (e.g., PE, PP) are discussed.
• Elevated temperatures may enhance plastic fragmentation and DOC leaching, indirectly influencing microbial degradation.

## Graphical abstract

**Figure:**
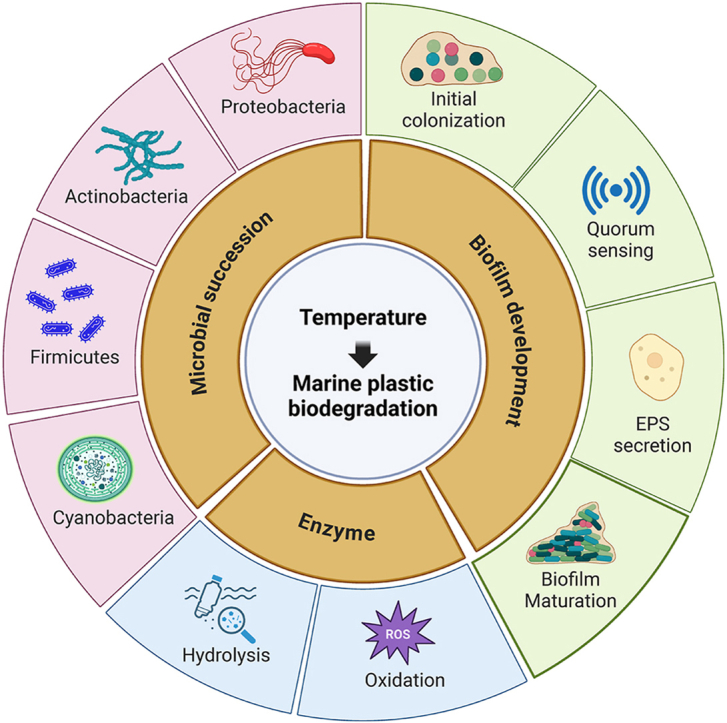

## 1 Introduction

Plastics are indispensable in our daily lives due to their durability and low cost. However, only about 9% of plastics are recycled, while an overwhelming 79% end up in landfills or the environment, and between 8 million and 11 million tons of plastic waste enter the ocean every year [1,2]. The biodegradation of plastic waste functions as a natural defense mechanism, mitigating the accumulation of synthetic pollutants in marine ecosystems. Biodegradable plastics have been promoted as a potential solution to the plastic pollution crisis. The global bioplastics production capacity is projected to increase significantly, from around 2.18 million tonnes in 2023 to approximately 7.43 million tonnes by 2028 [3].

The plastic degradation process in marine environments is affected by intrinsic properties (e.g., composition, material, structure, and existence form of plastics) as well as external environmental conditions (e.g., temperature, pH, salinity, microbial activity, water dynamics, hydrolysis, enzymes, and naturally occurring catalytic agents such as metal ions or photoreactive compounds) [4]. Among these, temperature is critical in shaping microbial dynamics, biofilm formation, plastic properties, and enzyme activity [5]. Plastics are distributed across diverse marine environments, from tropical waters to more extreme conditions. For instance, microplastics (MPs) have been detected in Arctic ice and the deep sea [6], highlighting the presence of plastic waste across a broad temperature range from −2 ​°C in polar regions to over 30 ​°C in tropical and temperate waters [7,8].

Moreover, climate change amplifies the impacts of marine plastic pollution through mechanisms such as ice melting, extreme weather events, and ocean acidification. For instance, the melting of Arctic sea ice is projected to release trillions of microplastic particles into marine systems within the next decade [9,10]. Rising ocean temperatures driven by climate change further complicate degradation processes by accelerating plastic fragmentation [11]. While moderate warming may enhance biodegradation within optimal biological ranges [11,12], extreme thermal conditions can inhibit microbial growth or denature key enzymes. These shifts complicate predictions of plastic fate in the marine environment. Thus, understanding the temperature dynamics across different marine regions is crucial for assessing the biodegradation potential of plastics in a warming world.

Research is increasingly focused on understanding the degradation mechanisms of both conventional and biodegradable plastics in marine environments, using *in situ* and laboratory-based biodegradation experiments. However, many investigations of marine plastic biodegradation remain limited to a single temperature range [13,14]. It lacks a broad comparison across the diverse thermal environments. As a result, critical gaps remain in our understanding of how different temperatures affect plastic degradation rates and microbial succession, particularly concerning seasonal variations and extreme conditions. Besides, studies overlook the long-term impacts of temperature fluctuations, given the prolonged persistence of plastics in marine environments. Addressing these gaps is essential for developing effective strategies to mitigate marine plastic pollution.

**Fig. 1:**
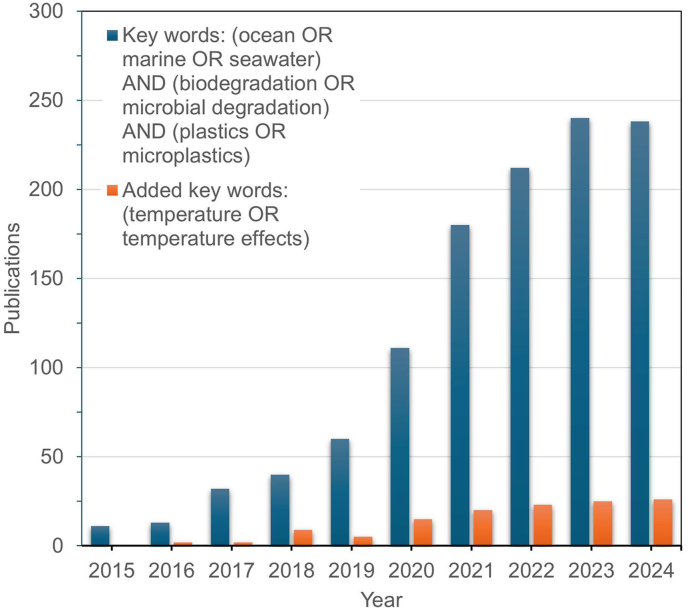
Publication trends of temperature effects on plastics marine biodegradation derived from literature search.

Using a systematic literature review methodology, we identified relevant publications by applying the search terms “ocean OR marine OR seawater” AND “biodegradation OR microbial degradation” AND “plastics OR microplastics” across all fields from the last 10 years (Fig. 1). To refine the focus, we further filtered articles by adding keywords of “temperature OR temperature effects”. The decreasing number of such publications highlights a significant research gap in this area (Fig. 1). To address this gap, this review summarizes and evaluates current research on the effects of temperature on marine plastic biodegradation, including key aspects like biofilm development, microbial succession, and enzyme-based depolymerization across varying temperature ranges. Furthermore, it addresses the broader environmental and ecological implications of temperature-mediated plastic degradation and proposes strategies for bio-mitigating the growing issue of plastic waste.

## 2 Temperature effects on biofilm development

Plastic pollution in marine environments was first recorded in the 1970s, particularly in the North Atlantic Ocean, where plastic particle concentrations reached up to 3500 pieces/km^2^ [15]. Plastics often undergo physical and chemical weathering before and after entering the ocean. These processes generate MPs (<5 ​mm) or nanoplastics (1 ​nm–1 ​μm), which have garnered significant attention due to their direct impacts on marine ecosystems and interactions with other contaminants [16]. Microorganisms colonize these MP surfaces, forming biofilms named plastisphere [17]. These biofilms enhance survival by supporting stable microbial consortia formation, horizontal gene transfer, nutrient accumulation, and protection from toxic substances [18]. Within the plastisphere, pioneer microorganisms colonize the plastic surface, initiating primary biodegradation by fragmenting the plastic into smaller pieces through the breakdown of polymer chains. Secondary colonizers then produce extracellular polymeric substances (EPS) to form more irreversible attachments of biofilm. These fragments are eventually mineralized by various enzymes secreted within the biofilms into CO_2_ [19]. The duration of each step varies; initial colonization occurs within minutes, while secondary biofilm development may take months, and final mineralization takes even longer [19].

**Fig. 2:**
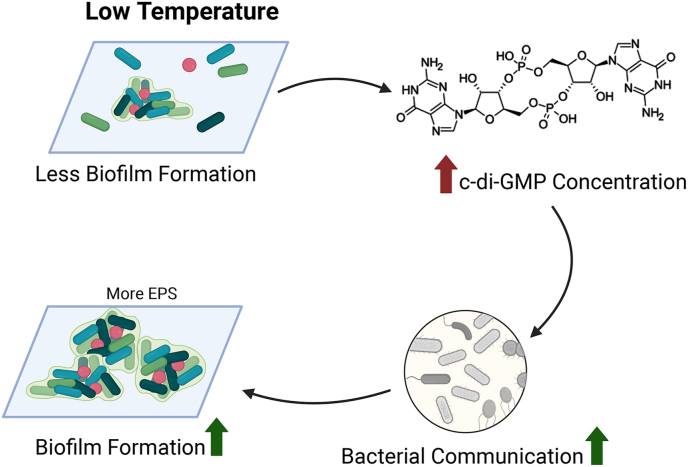
The mechanism of temperature on biofilm development at low temperature, adapted from Lin et al. [23].

Higher temperatures generally promote the early stages of biofilm formation by accelerating the growth of pioneer colonizers, such as members of the Gammaproteobacteria. These pioneer organisms stabilize the biofilm by releasing organic substrates, which support the development of more complex microbial communities [20]. Temperature not only influences physical processes like EPS secretion and biofilm maturation, but also modulates regulatory networks such as quorum sensing (QS). QS molecules, such as acyl-homoserine lactones, help coordinate microbial behavior during initial colonization and biofilm development. Elevated temperatures may accelerate QS signaling by increasing diffusivity or modifying signal stability [21]. Despite this, lower temperatures can enhance biofilm formation by accumulating cyclic di-GMP (c-di-GMP), a second messenger that regulates biofilm development, motility, and EPS production [22]. As illustrated in Fig. 2, increased c-di-GMP levels at low temperatures enhance bacterial communication and biofilm formation, creating a more stable microbial community on plastic surfaces [23]. For instance, cold-tolerant bacteria like *Erythrobacter* have been shown to colonize polystyrene (PS) and polyethylene (PE) in waters of 17 ​°C [24]. *Vibrio cholerae* can form more complex biofilms at lower temperatures (15 ​°C and 25 ​°C) than at 37 ​°C [25]. *Pseudoalteromonas* has been observed to produce more EPS at lower temperatures [26]. Similarly, Bisht et al. [27] demonstrated that *Pseudomonas aeruginosa* formed biofilms with higher biomass and more complex EPS matrices at 23 ​°C than at 37 ​°C. Notably, the study also revealed temperature-specific shifts in EPS-related protein expression, evidencing structural adaptations of the biofilm matrix in response to temperature variation. These findings indicate that lower temperatures may promote enhanced matrix production and protein heterogeneity in biofilms. Additionally, c-di-GMP plays a crucial role in flagellar regulation, downregulating motility and contributing to biofilm stability. Besides, temperature fluctuations can also influence the activity of extracellular attachment structures like flagella [5,28].

Besides, the diversity and structural complexity of mature biofilms on marine plastic debris are significantly influenced by environmental temperature. At moderate temperatures (approximately 20 ​°C–30 ​°C), microbial diversity on plastic surfaces tends to increase due to the enrichment of both mesophilic generalists and specialized degraders [29]. Conversely, at low temperatures (<5 ​°C), diversity typically decreases, with cold-adapted genera such as *Psychrobacter* and *Shewanella* dominating plastic-associated biofilms [30]. Elevated temperatures (>35 ​°C) may again reduce diversity by selecting for thermotolerant taxa such as *Pseudomonas* and *Rhodococcus*, which are known for their metabolic flexibility and ability to degrade hydrocarbons and plastics under thermal stress [31]. These temperature-driven shifts in microbial composition influence the structural stability and functional capacity of mature biofilms on plastic debris in marine environments. Various studies have explored the influence of environmental factors on plastic biofilm development, such as seasonality, temperature, and light. *In situ* experiments have provided valuable insights into the ecological dynamics of bacterial communities [32,33]. For instance, Pinnell and Turner [33] reported the critical impacts of seasonal variation on microbial diversity. However, investigating sole temperature effects on microbial dynamics remains challenging as field trials often have complex conditions such as different salinities and pH.

In summary, temperature plays an essential role in the formation and development of biofilms on marine plastic surfaces. While higher temperatures generally accelerate the early stages of biofilm formation, lower temperatures may enhance biofilm stability through mechanisms such as increased EPS production via c-di-GMP regulation. However, the full extent of temperature's influence on biofilm dynamics, particularly concerning long-term plastic degradation, remains insufficiently explored, especially given the complexity of microbial communities and the variety of plastic substrates. Further research is needed to unravel how these factors interact over time to influence the degradation process and to better understand how temperature fluctuations affect biofilm resilience and plastic breakdown in diverse marine environments.

## 3 Temperature-induced microbial succession

Temperature is a key driver of microbial succession in the degradation of plastics in marine environments, profoundly influencing the composition and activity of microbial communities. The four phyla, i.e., Proteobacteria, Bacteroidetes, Actinobacteria, and Cyanobacteria, were selected for focused discussion due to their consistent presence in plastic-associated marine biofilms and their diverse metabolic contributions to biodegradation processes. These phyla represent key ecological groups that respond differently to environmental stressors, including temperature, and thus serve as important models for understanding temperature-mediated biodegradation dynamics. For instance, the phylum Proteobacteria dominates initial colonization at ambient temperatures [18]. Besides, phyla Actinobacteria, Firmicutes, and Cyanobacteria further contribute to plastics biodegradation. In extreme environments, temperatures shape microbial communities differently: psychrophilic bacteria dominate in colder waters, while thermophiles take over in warmer regions, such as deep-sea black and white smokers [34]. Table 1 summarizes current studies on the temperature-dependent biodegradation efficiency of various plastics in marine environments, highlighting how temperature influences the succession and activity of microbial communities across different plastic types.

**Table 1:** Summary of studies on the temperature effects on biodegradation efficiency of various plastics in marine environments.

Plastic types	Form	Bacteria	Temperature	Duration	Location	Conditions	Result	Assessment	Reference
PHB	Powder, Film	*Shewanella*	4 ​°C, 15 ​°C, 25 ​°C, 30 ​°C, 37 ​°C, 50 ​°C, 60 ​°C	10 days	Yaizu, Suruga Bay, Japan	Lab	The clear zone on the P(3HB) medium was largest at 15 ​°C; strain grew well at 30 ​°C–37 ​°C.	Clear zone	[114]
PHBH, PLA, PBAT, PBS, PBSA, PCL	Film	*Glaciecola*, *Aestuariibacter halophilus*, *Pseudoalteromonas*	11 ​°C, 14 ​°C, 20 ​°C	1 month	Takasago harbor, Japan	Lab	PHBH and PCL films showed degradation, with biofilm formation being crucial for degradation efficiency.	Observation, 16S rRNA	[52]
PHBH, PP, Cellulose	Sheet, Flake	*Clostridiales*, *Gemmatales*, *Phycisphaerales*, *Chlamydiales*	25 ​°C (Room temperature)	148–195 days	Georgia, United States	Lab	Anaerobic sludge produced more methane with PHBH compared to cellulose. PHBH had variable CO_2_ production under aerobic conditions.	Gas evolution (CO_2_, Methane)	[115]
P(3HB)	Film	*Alcanivorax dieselolei* (MC1)	4 ​°C, 20 ​°C, 25 ​°C, 30 ​°C, 37 ​°C, 50 ​°C	5 days	Microbiota of cheeses, Japan	Lab	MC1 formed clear zones at 30 ​°C–37 ​°C, inactive at 4 ​°C. Nutrient-rich conditions suppressed P(3HB) degradation.	Clear zones	[116]
PHBH	Film	*Bacillus* sp., *Alteromonas*, *Psychrobacter*	4 ​°C	1 month	Deep-sea sediment, Japan	Lab	PHBH degraded by bacteria from deep-sea environments at low temperatures and high pressure.	Microbial composition	[99]
P(3HB)	Film	–	10 ​°C, 20 ​°C, 27 ​°C	4 weeks	Osaka South Port and Osaka Bay	Lab and field	PHAs biodegraded about 25%, higher temperature increased degradation.	BOD test (lab); Weight loss and molecular weight (field)	[80]
PET	Film	*Vibrio* sp. (bacteria), *Aspergillus* sp. (fungi)	25 ​°C, 35 ​°C, 45 ​°C	6 weeks	Bay of Bengal	Lab	Plastic bottle waste sample degraded 35% by bacterial strains and 22% by fungal strains. Best rate of degradation at 35 ​°C.	Weight loss, FTIR, SEM, XRD	[75]
P(3HB)	Film	*Nocardioides* sp. OK12	4 ​°C, 15 ​°C, 25 ​°C, 30 ​°C, 37 ​°C, 40 ​°C, 50 ​°C	7 days	Okinoshima beach, Japan	Lab	Optimum degradation at 30 ​°C. Biofilm formation enhanced degradation.	Clear zone, weight loss, FTIR, genetic analysis	[65]
PHAs	Film	*Bacillus* sp. JY14	20 ​°C, 30 ​°C, 37 ​°C, 42 ​°C	5 days	Marine soil, Korea	Lab	Highest PHB degradation at 30 ​°C (40% in liquid culture).	Weight loss, clear zone	[67]
PA4	Powder	*Pseudoalteromonas* sp. *Y-5*	15 ​°C	4 days	Marine environment	Lab	Maximum PA4-degrading activity observed after 4 days of incubation. The purified enzyme successfully hydrolyzed PA4 into gamma-aminobutyric acid oligomers.	Enzyme activity assays, mass spectrometry	[117]
PET	Bottle	Marine microbial community (Pseudoalteromonas 21%, Alteromonas 11%)	25 ​°C	150 days	Shuangyue Bay in Huizhou, China	Field	Light, high-pressure heat, and humid environments significantly affect degradation; high-salt environments have less effect. Ordinary cleaning processes are insufficient to remove inorganic substances.	SEM, Elemental Analysis, tensile test, viscosity	[118]
PE, LDPE	Film (from bottles, gloves)	*Pseudomonas aeruginosa, Halomonas venusta*	30 ​°C ​± ​2 ​°C	3 months		Lab	Light promotes the leaching of harmful additives; microbial activity, salinity, pH, and aeration significantly influence biodegradation. PE is more susceptible to degradation than LDPE.	SEM, FTIR, ICP-OES, GC/MS	[119]
PLA, PBS, PBAT, PBSC, PBAF	Film (0.15 and 0.5 ​mm thick)	Marine microbial community	Min: 11.6 ​°C (winter), Max: 22.8 ​°C (summer)	12 months	Pohang-si, South Korea	Field	Weight loss rate of polyesters directly affected by water temperature; average rate in summer (23 ​°C) is 1.9 times higher than in winter (12 ​°C). No surface modification was observed at 4 ​°C and 15 ​°C, holes started to appear on the surface, and these became larger and deeper with time at 25 ​°C.	SEM, molecular weight loss	[120]
PBS/PBAT	Rope	–	4 ​°C, 15 ​°C, 25 ​°C, 40 ​°C, 60 ​°C	18 months	Brest estuary	Lab	No significant loss in molecular weight at low temperatures; higher temperatures led to faster loss of properties.	Tensile test, Molecular weight loss, SEM	[121]
PHB, PBSe, PBSeT, LDPE	Film	–	20 ​°C (lab), 12 ​°C–30 ​°C (field)	331 days	Seawater taken at Seccheto, Isola d'Elba, Italy	Lab and field	Biodegradation half-life varies significantly by climate, habitat, and material; LDPE showed no biodegradation.	CO_2_ release	[122]
PE, PLA, tire particles	Particles	Marine microbial community	5 ​°C and 25 ​°C	60 days	Pingdingshan, China	Lab	Temperature influences catalase and neutral phosphatase activities; tire particles increase microbial diversity and alter community structure.	PCR, SEM, FTIR, enzyme activity	[86]
PCL, PBS, PBAT	Film	*Vibrio* species dominant	Lab: room temp, Field: 3 ​°C (winter), 27 ​°C (summer)	1 year	Odo 1-ri, Heung-hae-eup, Buk-gu, Pohang City, Gyeongsangbuk-do, South Korea	Lab and field	PCL degraded at the rate of 89 ​μm/month in a coastal marine environment; ranking of decomposition rates was PCL ​> ​PBS ​> ​PBAT. After 12 months, tensile strength of PCL decreased to 15 ​MPa and elongation to almost 0.	Molecular weight loss, tensile test, SEM	[123]
PE and PET	Film	*Pseudomonas, Bacillus, Vibrio*	26 ​°C (room temperature)	4 weeks	Huiquan Bay, Qingdao, China	Field	Molecular weight loss and surface erosion observed in both PET and PE samples.	16S rRNA, SEM, FTIR, GPC, XRD, HPLC-MS	[124]
PCL	Film	*Pseudomonas pachastrellae*	4 ​°C, 20 ​°C, 25 ​°C, 30 ​°C, 37 ​°C, and 40 ​°C	1 week	Okinoshima coastal water, Chiba, Japan	Lab	Strain TKCM 64, closely related to *Pseudomonas pachastrellae*, degraded PCL film at a rate of 1.39 ​± ​0.09 mg/(cm^2^·day); hydrolytic activity induced by PCL and its hydrolysate 6-hydroxyhexanoic acid.	PCR, GC, FAME	[43]
PLA, PVA/starch blends, LDPE	Bag strip		22.86 ​°C ​± ​4.28 ​°C	6 months	Hong Kong, China	Field	All marine plastic samples show notable biofouling growth and fragmentation. PLA and PVA/Starch blends show larger mass losses by 23%–100% than the LDPE.	Weight loss, SEM, FTIR	[88]

FAME, Fatty acid methyl esters; FTIR, Fourier-transform infrared spectroscopy; GC/MS, Gas chromatography/mass spectrometry; GPC, Gel permeation chromatography; HPLC-MS, High-performance liquid chromatography-mass spectrometry; ICP-OES, Inductively coupled plasma optical emission spectroscopy; LDPE, Low-density polyethylene; P(3HB), Poly(3-hydroxybutyrate); PA4, Polyamide 4; PBAF, Poly(butylene adipate-co-furanoate); PBAT, Poly(butylene adipate-co-terephthalate); PBS, Polybutylene succinate; PBSA, Poly(butylene succinate-co-butylene adipate); PBSC, Poly(butylene succinate-co-carbonate); PBSe, Polybutylene sebacate; PBSeT, Polybutylene sebacate co-butylene terephthalate; PCL, Polycaprolactone; PCR, Polymerase chain reaction; PET, Polyethylene terephthalate; PHAs, Polyhydroxyalkanoates; PHB, Polyhydroxybutyrate; PHBH, Poly(3-hydroxybutyrate-co-3-hydroxyhexanoate); PLA, Polylactic acid; PP, Polypropylene; PVA, Polyvinyl alcohol; SEM, Scanning electron microscope; XRD, X-ray diffraction.

### 3.1 Proteobacteria

As one of the largest bacterial phyla, Proteobacteria encompasses a variety of species with notable plastic-degrading capabilities in marine ecosystems due to their metabolic diversity and adaptability to various environmental conditions. A prominent example from the Betaproteobacteria class is *Ideonella sakaiensis,* primarily recognized for its ability to degrade polyethylene terephthalate (PET) at 30 ​°C [35].

Among the Gammaproteobacteria, several marine-adapted species stand out for their plastic-degrading abilities. For instance, *Alcanivorax* species, a vital member of the hydrocarbonoclastic clade, have been shown to contribute significantly to the degradation of PE [36], polyhydroxybutyrate (PHB) [37], and PS [38] under moderate temperatures. Notably, it was reported that higher ambient temperatures can enhance the release of fragments and degradation by-products during the breakdown of PE, Polylactic acid (PLA), and Polyhydroxyalkanoates (PHAs) by *Alcanivorax* [39]. Studies on oil-degrading bacteria have also isolated *Alcanivorax* species from cold marine environments [40]. Its cold adaptation aids plastic degradation; for example, polypropylene (PP) demonstrated more efficient breakdown at 10 ​°C than at 20 ​°C in mesopelagic environments [41].

Another member of the Gammaproteobacteria, *Pseudomonas*, can survive at temperatures from 4 ​°C to 42 ​°C [42]. It has been shown to degrade various plastics in marine environments, such as polycaprolactone (PCL) in coastal waters [43,44]. Its metabolic versatility, particularly in degrading PET, has been observed under deep-sea conditions with high pressure and low temperatures [45]. Moreover, *Pseudomonas aeruginosa* displayed the highest cell abundance on PE and degradation activity at 23 ​°C than at 44 ​°C [46]. Mixed cultures of *Pseudomonas aeruginosa* and *Brevibacterium* sp. degraded low-density polyethylene (LDPE), with a weight loss of 5.22% after 30 days at 25 ​°C and decreased at higher temperatures, with 4.14% loss at 30 ​°C and only 2.24% at 35 ​°C [47]. These findings indicate that *Pseudomonas* species may achieve optimal plastic degradation at moderate temperatures around 25 ​°C, with their activity declining as temperatures increase.

**Fig. 3:**
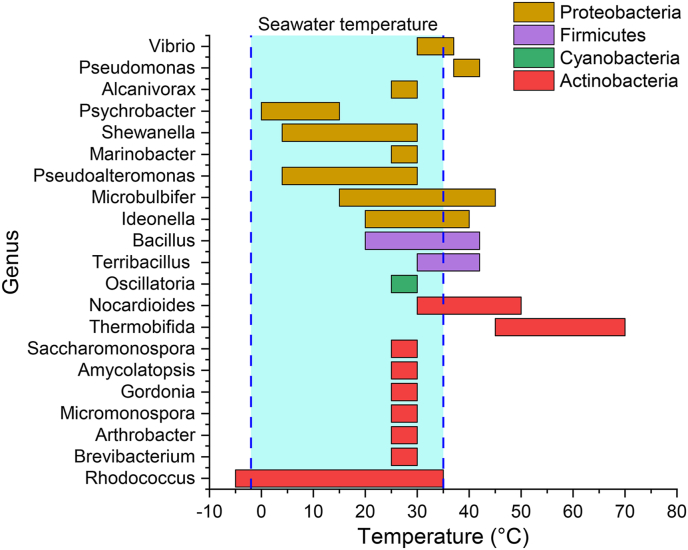
Temperature ranges for marine plastic-degrading bacteria across different phyla.

The genus *Vibrio* demonstrates varied responses to temperature, with some species thriving between 30 ​°C and 37 ​°C (Fig. 3), while others exhibit more complex or even inverse reactions [48]. Notably, studies reveal that *Vibrio* species form more substantial biofilm biomass at 25 ​°C than at higher temperatures on LDPE, PP, and PS [49]. Besides, biofilm formation on plastics enhances plastics degradation and increases their dispersal across marine ecosystems. Given the pathogenic nature of many *Vibrio* species and their ability to travel on MPs, they pose ecological and public health risks [50]. As global ocean temperatures rise, *Vibrio* may present increased challenges, with warmer conditions potentially amplifying both plastic degradation and the spread of pathogenic strains [48].

*Marinobacter* species, belonging to the Alteromonadales order, demonstrate strong capabilities in degrading PE across a wide temperature range (4 ​°C–30 ​°C) in marine environments [51]. Additionally, Alteromonadales are identified as primary degraders of PHAs in biofilms on plastic films submerged in seawater. A study shows that increasing seawater temperatures from 11 ​°C to 20 ​°C correlates with enhanced PHA degradation [52].

In cold environments, psychrophilic bacteria like *Psychrobacter* sp. *NJ228*, isolated from Antarctic sea ice, play a crucial role in marine plastic biodegradation by producing cold-adapted enzymes such as laccase. This enzyme shows optimal activity at temperatures between 10 ​°C and 20 ​°C while still retaining functionality at 0 ​°C [53]. *Psychrobacter* and other psychrophilic bacteria thrive in cold conditions by modifying the composition of their cell membranes. Specifically, they increase the proportion of unsaturated and short-chain fatty acids, which helps maintain membrane fluidity at low temperatures. This adaptation preserves cell integrity and allows efficient nutrient transport and enzyme activity [54]. Moreover, psychrophilic bacteria also produce cold-shock proteins (Csp) to stabilize RNA structures at low temperatures and ensure stable protein synthesis and enzyme production [55]. Thus, maintaining membrane fluidity and the production of Csp is crucial for sustaining plastic biodegradation by psychrophilic bacteria in cold marine environments.

In addition to these adaptations, insights from hydrocarbon-degrading bacteria provide further reference for understanding plastic biodegradation under extreme conditions [56]. Cold-adapted bacteria express cold-active enzymes such as oxygenases, esterases, and PETases, which may facilitate plastic depolymerization [57]. These microbes also produce EPS and antifreeze proteins to enhance biofilm formation, promote adhesion to plastic surfaces, and protect cells from freezing damage [57,58]. In deep-sea environments, the expression of hydrocarbon degradation gene clusters and the use of alternative respiration pathways, such as nitrate or sulfate reduction, may also support microbial plastic degradation under high-pressure and oxygen-limited conditions [59].

### 3.2 Actinobacteria

Actinobacteria, a prominent phylum recognized for their role in plastic biodegradation, demonstrate a wide range of temperature adaptability. These bacteria are also renowned for their ability to break down petroleum hydrocarbons alongside plastics [60]. In addition, marine-derived Actinobacteria not only biodegrade conventional plastics like LDPE and PS but can also utilize these plastics as a carbon source to produce biodegradable PHA bioplastics [61]. This adaptability, particularly under mesophilic conditions, underscores the ecological versatility of Actinobacteria in facilitating marine plastic biodegradation.

Within the family of *Nocardiaceae*, the genus *Rhodococcus* stands out due to its remarkable temperature adaptability. It can grow across a wide temperature range, from −5 ​°C to 50 ​°C, optimally at 37 ​°C [62]. Besides, the ability of *Rhodococcus* to degrade long-chain alkanes and other recalcitrant compounds further reinforces their significance in regulating marine biogeochemical cycles [63]. In cold environments, *Rhodococcus* species continue to play a vital role. *Rhodococcus* sp. *JG3*, isolated from the Antarctic Dry Valley permafrost, has been observed to grow at temperatures as low as −5 ​°C. This cold tolerance is primarily attributed to gene expression adaptations that enhance energy production and redox homeostasis under cold stress [63]. Similarly, *Rhodococcus fascians* from Antarctica has been reported to produce bioemulsifiers that enhance the biodegradation of hydrocarbons like hexadecane and biphenyl between 4 ​°C and 35 ​°C [60].

*Nocardiopsis*, another critical genus from *Nocardiaceae*, has been identified for its efficiency in plastic degradation under mesophilic conditions. For instance, *Nocardiopsis* isolated from marine sediments was found to excrete extracellular PHB depolymerase and grow efficiently on PHB and its copolymers as the sole carbon source at 30 ​°C [64]. Additionally, it demonstrated significant degradation of poly(3-hydroxybutyrate) [P(3HB)] films and formed biofilms on P(3HB) and PP surfaces, with optimal growth occurring at 30 ​°C, although it did not survive at temperatures above 50 ​°C [65].

### 3.3 Firmicutes

Within the phylum Firmicutes, numerous species, particularly those in the *Bacillaceae* family, are known for their plastic-degrading abilities at 25 ​°C–37 ​°C (Fig. 3). The genus *Bacillus* is widely studied. For instance, *Bacillus infantis* PD3 achieved a remarkable 98.71% degradation of PHB film within 5 ​day ​at 37 ​°C in a mineral medium [66]. Similarly, *Bacillus* sp. JY14, a thermotolerant strain, can degrade PHB film from 20 ​°C to 42 ​°C, with the highest efficiency observed at 30 ​°C [67]. This adaptability underscores its potential for plastic degradation in environments with fluctuating temperatures. Alongside *Bacillus*, *Terribacillus* shows notable plastic degradation abilities, demonstrating activity on PBAT and PCL and degrading PBS at 42 ​°C. The clear zone expansion was slower at 42 ​°C than at 30 ​°C and 37 ​°C, suggesting higher efficiency at moderate temperatures [68].

### 3.4 Cyanobacteria

Cyanobacteria, a diverse group of photosynthetic bacteria, play an increasingly recognized role in marine plastic degradation. *Oscillatoria subbrevis* is particularly effective at degrading plastics from 25 ​°C to 30 ​°C [69]. Additionally, filamentous cyanobacteria such as *Leptolyngbya*, *Limnothrix*, *Phormidium*, *Prochlorothrix*, and *Rivularia* colonize plastic debris in regions like the Great Pacific Garbage Patch and the North Atlantic [70]. Seasonal shifts affect biofilm composition and activity; for instance, it is reported that *Pseudophormidium* tends to dominate biofilms on PS particles in warmer months, while *Synechococcus* becomes more prevalent in cooler conditions, influencing carbon and nutrient cycling in marine environments [19]. With rising ocean temperatures, the role of Cyanobacteria in plastic degradation and nutrient regulation is likely to expand, potentially impacting marine food webs and biodiversity.

Taking all this together, temperature is a crucial factor in microbial succession during marine plastic degradation, influencing which phyla dominate and how effectively they degrade plastics. Proteobacteria initiate the breakdown at moderate temperatures, while Actinobacteria, Firmicutes, and Cyanobacteria continue to serve in subsequent stages, adapting to varied temperatures and further facilitating degradation. In extreme conditions, psychrophilic and thermophilic bacteria drive degradation within cold and warm environments, respectively. Seasonal shifts also alter the biofilm composition of Cyanobacteria, with microbes like *Pseudophormidium* and *Synechococcus* affecting nutrient cycles. Importantly, elevated temperatures can enhance or suppress degradation rates depending on the optimum temperature of key degraders, and may facilitate the proliferation of opportunistic pathogens like *Vibrio* on plastic surfaces. Beyond individual functions, temperature also modulates ecological interactions among these groups. In warm marine environments, Cyanobacteria may enhance local oxygen concentrations, promoting aerobic degradation pathways facilitated by Proteobacteria. Conversely, in colder and deeper marine zones, Actinobacteria could maintain community function when other taxa become less metabolically active. These inter-phyla interactions, shaped by temperature-driven changes in enzymatic activity, community succession, and metabolic cooperation, may determine the overall efficiency of plastic biodegradation across different temperatures. These insights underscore how temperature mediates microbial succession and plastic biodegradation efficacy in marine ecosystems.

## 4 Enzyme-based plastic depolymerization under different temperatures

**Fig. 4:**
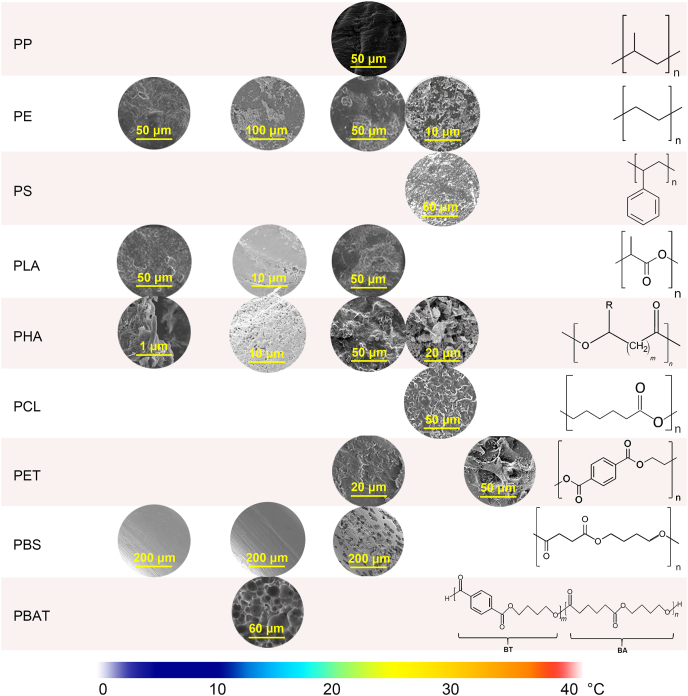
SEM images of plastics showing morphological changes by microbial degradation under different temperatures. Images are adapted from references [39,43,67,75,86,107,115,[119], [120], [121],^124^,^125^].

Enzymes play a central role in breaking down complex polymer chains into smaller fragments, facilitating microbial assimilation and plastic degradation. The efficiency of this process is strongly influenced by temperature, which affects enzyme structure, flexibility, and catalytic performance [71]. Hydrolytic enzymes, such as PETases, cutinases, and lipases, tend to perform best at moderate temperatures. Under these conditions, enhanced active site dynamics improve the cleavage of ester bonds in plastics like PET and PLA. In contrast, oxidative enzymes, including monooxygenases, peroxidases, and cytochrome P450s, initiate the degradation of non-hydrolyzable plastics such as PE and PS by producing reactive oxygen species (ROS), and these enzymes often maintain functionality over a broader temperature range. To improve clarity and accessibility for a diverse readership, we present this section according to plastic type rather than enzyme class. Plastics are broadly grouped into two categories: hydrolyzable plastics, including PET, PCL, PLA, and PHAs, and non-hydrolyzable plastics, such as PE, PP, and PS. For each category, we summarize the key enzymes involved in depolymerization (Table 2) and describe the morphological changes observed under different temperature conditions (Fig. 4).

**Table 2:** Key enzymes in marine plastic degradation and their temperature ranges.

Enzyme	Plastic type	Observed degradation temperature	Role in degradation
Cutinase	PET, PCL	30 ​°C–70 ​°C	Hydrolyzes ester bonds in PET, breaking it down into terephthalic acid and ethylene glycol.
PHA depolymerase	PHAs	4 ​°C–40 ​°C	Depolymerizes PHAs by breaking ester bonds, producing hydroxyalkanoic acids.
Proteinase K	PLA	40 ​°C–60 ​°C	Hydrolyzes ester linkages in PLA, breaking it down into lactic acid monomers.
Lipase	PCL, PHAs, PLA	25 ​°C–37 ​°C	Breaks down PCL by hydrolyzing ester bonds, particularly at interfacial areas.
Laccase	PS, PE, PLA	30 ​°C–50 ​°C	Catalyzes the oxidation of the polymer backbone, facilitating microbial attack.
Alkane hydroxylase	PP, PE	25 ​°C–40 ​°C	Initiates oxidation of hydrocarbons, aiding in the breakdown of PP and PE.
Monooxygenase	PE, PP, PS	30 ​°C–35 ​°C	Generates ROS, oxidizing C–C bonds for further breakdown.
Ring-hydroxylating dioxygenase	PS	25 ​°C–35 ​°C	Cleaves the aromatic ring structure in PS, leading to partial degradation.

### 4.1 PET

PET is among the most widely produced thermoplastics, extensively used in packaging and textile production due to its durability and resistance to degradation. Crystallization initiates at 70 ​°C, theoretically providing optimal conditions for degradation by balancing chain mobility and enhanced enzyme activity [72]. Although PET is chemically hydrolyzable, it is not readily biodegradable under ambient marine conditions and typically requires elevated temperatures and specialized enzymes such as PETase and MHETase for effective depolymerization. However, achieving these conditions in marine environments is challenging, as the limited prevalence of plastic-degrading enzymes and pelagic conditions suggests a lower degradation potential than in terrestrial ecosystems [73]. In colder regions like the Arctic, where temperatures are near 0.5 ​°C, PET degradation proceeds extremely slowly, requiring approximately 162 years for 50% depolymerization. Conversely, in tropical regions at around 35 ​°C, PET achieves 50% degradation in 4.5 years [74].

PET depolymerization typically undergoes three stages: First, PET is broken down into two primary intermediates by PETase: mono(2-hydroxyethyl) terephthalate (MHET) and bis(2-hydroxyethyl) terephthalate (BHET). These intermediates are then further degraded into terephthalic acid (TPA) and ethylene glycol (EG) through the action of enzymes such as MHETase and cutinase, which cleave the ester bonds [71]. Finally, mineralization occurs, converting intermediates into non-toxic by-products, though this process may be inhibited in marine environments due to factors such as salinity and low temperatures [71].

One notable enzyme in PET depolymerization is *Is*PETase, secreted by *Ideonella sakaiensis*. It exhibits a high degradation capacity at moderate temperatures (about 30 ​°C). It aligns with SEM observations, which show no signs of degradation on PET films at lower temperatures; however, at 36 ​°C, the PET surface loses smoothness, with visible cracks observed [75]. This thermal sensitivity is primarily attributed to its structural features. *Is*PETase possesses a relatively flexible β-sheet core and surface-exposed loops surrounding the active site, which, while facilitating substrate binding at lower temperatures, compromise structural rigidity at higher temperatures [76]. As the temperature increases, these flexible regions are prone to partial unfolding, leading to misalignment of the catalytic triad and disruption of hydrogen bonding networks essential for enzymatic activity [76]. Additionally, *Is*PETase has a relatively low melting temperature (about 45 ​°C), making it susceptible to thermal inactivation through destabilization of key active site residues and surrounding hydrophobic cores [77]. These molecular characteristics explain the observed decline in activity around 36 ​°C.

Furthermore, MHETase exhibits optimal activity at moderate temperatures (30 ​°C–35 ​°C) when transforming the PET oligomer to TPA and EG [71]. Besides, carboxylic ester hydrolases from *Pseudomonas aestusnigri* have been identified to hydrolyze PET films at around 30 ​°C [78]. These limitations highlight the delicate balance between enzyme flexibility and thermal stability, underscoring the need for protein engineering approaches to enhance enzymatic resilience and broaden the operational temperature range for efficient PET degradation. Advances in enzyme optimization, such as those demonstrated by Son et al. [77], offer promising pathways for improving PET biodegradation under diverse environmental conditions.

### 4.2 PCL

PCL is a biodegradable plastic with a low melting point (Tm, ​58 ​°C–63 ​°C), a low glass transition temperature (Tg, ​about ​−65 ​°C), and high crystallinity, leading to its widespread use in biomedical applications. Optimal degradation of PCL typically occurs between 30 ​°C and 60 ​°C [79]. Compared to other bioplastics such as PLA, PBS, and Poly(butylene succinate-co-butylene adipate) (PBSA), PCL demonstrates faster enzymatic degradation rates in marine environments due to its lower Tm and Tg [80]. PCL has also been used as a model substrate to replace PET due to its less complex structure. Suzuki et al. [43] observed that the pristine PCL film was very smooth and became much rougher at 30 ​°C. Several enzymes, including esterases, lipases, and carboxylesterases, are involved in PCL hydrolysis by catalyzing the breakdown of the ester bonds. For instance, lipases from mesophilic organisms such as *Pseudomonas aeruginosa* typically show optimal activity between 30 ​°C and 40 ​°C, with peak catalytic efficiency at 37 ​°C [81]. Additionally, new strategies, such as incorporating marine-derived proteins into PCL biocomposites, have shown higher degradation rates at 20 ​°C over 56 days, as protein fillers serve as nutrients for microorganisms [82].

### 4.3 PLA

PLA, one of the most widely used biodegradable plastics, is popular in packaging and medical devices. Its degradation is primarily facilitated by enzymes such as Proteinase K, lipases, esterases, and cutinases, which hydrolyze ester bonds [83]. At higher temperatures (40 ​°C–60 ​°C), enzyme activity increases, accelerating hydrolysis due to improved enzyme–substrate interactions and greater flexibility in PLA chains. While PLA degrades rapidly under composting conditions at 60 ​°C, it remains recalcitrant in cooler marine environments. For example, Bagheri et al. [84] observed no significant mass loss of PLA after 400 days in seawater at 25 ​°C. Chamas et al. [85] estimated that, although PLA degrades about 20 times faster than HDPE on land, it also has a similarly limited degradation in marine environments. Other studies also found no significant morphological changes on PLA surfaces between 4 ​°C and 25 ​°C after two months [39,86]. Royer et al. [87] reported no signs of PLA degradation after prolonged 428 days in the colder waters off California (13 ​°C–23 ​°C), indicating slower depolymerization in cold marine environments.

Notably, Cheung and Not [88] observed a different outcome, with PLA samples disintegrating within 1 ​month ​at an average temperature of 23 ​°C in Hong Kong's coastal waters, outperforming LDPE (Table 1). They hypothesized that the warmer and more stable seawater in Asian subtropical regions may create a more favorable environment for PLA hydrolysis compared to European temperate or Mediterranean climates.

Moreover, Proteinase K emerges as a critical enzyme for enhancing hydrolysis. It is particularly effective under optimal conditions around 37 ​°C [83], and its efficiency and thermal stability in PLA hydrolysis have been documented at temperatures near 50 ​°C [89,90]. Embedding Proteinase K in PLA matrices has been proven to significantly accelerate degradation, achieving a 78% weight loss of PLLA films within 96 ​h [91]. He et al. [92] also demonstrated substantial weight loss in PLA copolymers exposed to Proteinase K, underscoring the enzyme's potential to enhance PLA biodegradation in marine environments.

### 4.4 PHAs

Bioplastic PHAs synthesized naturally by organisms are considered superior to other bioplastics due to their complete biodegradability in marine ecosystems [93]. PHAs exhibit significant biodegradability across a wide temperature range in various marine environments, including coastal, shallow-water, and deep-sea regions [94]. The average degradation rate of PHAs in marine settings is estimated to be between 0.04 and 0.09 mg/(day·cm^2^), suggesting that an 800 ​μm thick PHA-based water bottle could take approximately 1.5–3.5 years to be fully biodegraded [95].

Early stages of PHAs degradation involve enzymatic breakdown into smaller oligomers, primarily mediated by enzymes such as PHA depolymerases (PhaZ), carboxylesterases, and lipases [96]. The enzymatic systems differ between short-chain-length PHAs (scl-PHAs) and medium-chain-length PHAs (mcl-PHAs). Scl-PHA depolymerases operate optimally across a broad temperature range of 30 ​°C–90 ​°C, while mcl-PHA depolymerases are most effective between 35 ​°C and 70 ​°C [97]. P(3HB), the most common PHA produced by microorganisms, exhibits optimal degradation between 37 ​°C and 55 ​°C [94].

The enzyme efficiency of PHA degradation generally increases with higher temperatures as enzymes become more flexible and effectively cleave ester bonds in PHA polymers. Several studies observed abundant bacterial cells attached to plastic surfaces. The surface became porous, and pore size increased along with incubation time and temperature (Fig. 4). For instance, Deroiné et al. [98] found that polyhydroxybutyrate-co-hydroxyvalerate (PHBV) degraded significantly faster in seawater at 40 ​°C compared to 4 ​°C or 25 ​°C. In contrast, it is reported that PHA degradation slows considerably in colder marine environments (below 15 ​°C) as reduced enzymatic activity and lower microbial metabolism inhibit the breakdown process [99,100].

A distinctive feature of PHA is its release of by-products, such as dissolved organic carbon (DOC) and MPs, as demonstrated in short-term microcosm studies. Temperature notably influences the extent of this release. Studies indicate that PHAs become increasingly porous (Fig. 4), resulting in a more significant release of MP fragments and DOC than PLA or PE at temperatures of 4 ​°C, 15 ​°C, and 22 ​°C after 60 days; higher temperatures further accelerate this release [39]. PHAs also release oxidized EPS-related fragments within a few days [96]. These findings highlight temperature's critical role in accelerating PHA degradation rates and influencing the quantity and size of MP particles entering the marine environment.

### 4.5 PE and PP

PE and PP are among the most widely produced plastics globally, highly valued for their durability and chemical stability. This stability primarily results from their non-hydrolyzable C–C backbone and the absence of functional groups, such as ester bonds, which make them inherently resistant to microbial degradation and hydrolysis [101]. For instance, the PP surface remained unchanged after incubation for 195 days at 24 ​°C (Fig. 4). In marine environments, the biodegradation of PE and PP depends on the formation of reactive oxygen species (ROS) generated by enzymes such as catalase-peroxidase, catalase, superoxide dismutase, cytochromes P450, and alkane monooxygenase (AlkB) [102]. These ROS facilitate initial oxidative changes within the polymer structure, creating weak points in the C–C backbone by forming hydroxyl and carbonyl groups to enhance susceptibility to microbial degradation. These enzymes produce modified fragments that are smaller, more accessible, and more readily biodegradable by microbial consortia [103]. A representative marine species is *Alcanivorax borkumensis*, which can utilize ROS to oxidize the PE and PP's surface and improve access for subsequent enzymatic assimilation [36,41]. Typically, elevated temperatures can accelerate the effectiveness of these oxidative and microbial processes in marine environments [104]. For example, cracks and pitting appeared on the PE surface at lower temperatures. More significant morphological changes, such as small holes and grooves, appeared under higher temperatures (30 ​°C) (Fig. 4).

However, the overall temperature-mediated degradation rate of PE and PP in marine environments remains limited, as most studies are conducted over relatively short observation periods, which overlook the long-term temperature effects. Further research is needed to establish a more comprehensive understanding of how temperature variations over extended periods impact the biodegradation pathways of these recalcitrant plastics in marine ecosystems.

### 4.6 PS

PS is another commonly produced plastic that poses significant challenges for degradation due to its aromatic ring structure and C–C backbone. Similar to PE and PP, ROS initiates the enzymatic degradation of PS. However, this process requires substantial energy input and progresses slowly, even under favorable conditions. Enzymes, such as cytochrome P450s, alkane hydroxylases, and monooxygenases, contribute to the degradation of PS's C–C backbone [105]. Moreover, the aromatic ring in PS makes enzymatic depolymerization particularly challenging compared to PE and PP. Only limited ring-hydroxylating dioxygenases in certain marine bacteria are reported to cleave PS side chains and oxidize the aromatic ring, breaking it into smaller, more manageable compounds [105,106].

Based on current knowledge, predicting an optimal temperature for the enzymatic degradation of PS remains challenging due to the limited studies on this topic and the incomplete understanding of the biochemical mechanisms associated with specific enzymes. Only a few studies have shown the potential of PS marine biodegradation. Syranidou et al. [107] utilized marine consortia to degrade PS films at 28 ​°C and found the signs of biodegradation, such as fissures, cracks, and roughness on the PS film surface after 6 months (Fig. 4). In addition, indigenous marine bacteria isolated from mangrove PS pollutants show a 2.66%–7.73% degradation rate at 28 ​°C over one month [108]. Cooler marine temperatures (15 ​°C–25 ​°C) and enzyme efficiency of PS biodegradation [50,109].

In brief, the degradation of hydrolyzable plastics primarily occurs through hydrolysis at moderate temperatures, facilitated by enzymes such as cutinases, lipases, and depolymerases. This process results in notable morphological changes, including surface erosion and increased porosity, particularly in PHAs. In contrast, non-hydrolyzable plastics with a resilient C–C backbone rely on enzymes such as monooxygenases and peroxidases to generate ROS that initiate oxidative modifications, breaking the polymer into monomers for further enzymatic breakdown. These polymers generally retain a smoother surface after degradation. Due to their slow biodegradation rates and limited biodegradability, the effects of temperature on this process are not readily apparent. Moreover, elevated temperatures typically accelerate degradation rates and increase the release of MPs and DOC from PHAs. Although lower temperatures may slow degradation, it still occurs, leaving persistent fragments in the marine environment. Current studies on the long-term effects of temperature on marine plastic biodegradation remain limited, which restricts a comprehensive understanding of how temperature variations may impact degradation rates.

## 5 Conclusion and future developments

This study underscores the critical role of temperature in marine plastic biodegradation, impacting biofilm formation, microbial succession, and degradation pathways. Findings reveal that higher temperatures generally stimulate early biofilm formation, enhancing microbial colonization. In specific cases, lower temperatures promote EPS production to stabilize biofilms for cold-tolerant bacteria. Hydrolyzable plastics, such as PHAs, degrade most effectively at moderate temperatures due to optimal enzymatic activity, resulting in significant morphological alteration. On the other hand, non-hydrolyzable plastics depend on oxidative modifications initiated by ROS to enhance their susceptibility to microbial degradation. Temperature has minimal influence on this process due to the limited biodegradation rate of these plastics.

Despite these insights, several research gaps persist. Current experiments are often limited to short observation periods of just a few months, while the complete biodegradation of plastics can span years, thereby restricting our understanding of the long-term impacts of temperature on degradation dynamics. Besides, field studies also struggle to isolate the sole effects of temperature on degradation, as real-world conditions involve multiple interacting environmental factors. Furthermore, while many bacteria exhibit plastic-degrading potential, optimal activity is typically observed at temperatures between 25 ​°C and 30 ​°C, with specific resilient strains required to thrive at lower temperatures. It is also important to consider whether the temperatures in known marine plastic sink zones align with the optimal ranges for biodegradation. For example, the North Pacific Subtropical Gyre and other ocean gyres exhibit moderate temperatures (approximately 23 ​°C–28.5 ​°C) [110], which may support enzymatic activity for certain plastic-degrading microbes. In contrast, cold environments like Arctic ice or deep-sea sediments fall well below optimal microbial activity temperatures, potentially slowing degradation processes. These spatial disparities highlight the need for region-specific strategies to address plastic accumulation.

In addition, it is well established that plastic degradation begins with the secretion of extracellular enzymes capable of depolymerizing high-molecular-weight polymers; however, most current studies have focused on enzyme activity after secretion, with limited attention to how temperature influences the secretion process itself [111]. For example, in other microbial systems such as *Aspergillus niger*, temperature has been shown to significantly affect the transcription and secretion of extracellular cellulases [112]. However, similar mechanisms regulating plastic-degrading enzymes remain largely unexplored. Moreover, temperature likely affects the production and scavenging of ROS, which are involved in the degradation of non-hydrolyzable plastics. Future research should investigate how temperature influences enzyme structure, function, and expression, particularly in relation to oxidative pathways. These efforts will be critical to advancing our understanding of temperature-mediated plastic biodegradation in marine and other natural environments.

While this review emphasizes temperature as a primary driver of microbial plastic degradation, it is important to recognize that degradation in marine environments is rarely governed by temperature alone. Environmental factors such as salinity, pH, nutrient availability, oxygen levels, and hydrodynamics also influence microbial community composition, enzyme activity, and the overall degradation process. These variables often interact synergistically with temperature, shaping both the rate and extent of biodegradation. A more holistic understanding of these combined effects is essential for accurately predicting plastic fate in complex marine ecosystems. Future studies should integrate multifactorial experimental designs to better simulate real-world conditions and inform effective mitigation strategies.

For future studies, integrating microbial genetic databases and machine learning offers significant potential for optimizing plastic degradation across diverse thermal conditions. Microbial databases can identify key plastic-degrading enzymes and metabolic pathways, revealing adaptive functions that enable microorganisms to thrive in temperature extremes, such as cold polar waters or increasingly warm tropical seas. These insights can guide the design of microbial consortia tailored to degradation processes that remain efficient under varying thermal regimes. Machine learning can complement these efforts by predicting how enzymes interact with their environments and optimizing microbial assemblages to enhance biodegradation under temperature fluctuations. For example, Lin and Zhang [113] developed a machine learning model informed by a meta-analysis of 74 polymers, using both literature and experimental data, to predict aerobic biodegradation in aquatic environments with high accuracy. By modeling enzyme stability and activity, machine learning can inform enzyme selection or engineering for superior performance in both cold and warm marine conditions. Tools like AlphaFold can further refine enzyme design, enabling the development of thermally resilient enzymes suited for degrading specific plastic types in distinct marine environments.

Furthermore, as climate change drives shifts in ocean temperatures and disrupts ecosystems, these advancements will be critical for developing resilient biodegradation strategies. Warmer oceans may enhance enzymatic activity and microbial growth in some regions, while polar and deep-sea environments demand innovations to overcome the constraints of low temperatures. By addressing these challenges, these temperature-focused solutions can contribute to more effective plastic pollution management in a rapidly changing climate, safeguarding marine ecosystems from the growing threat of plastic waste.

## CRediT authorship contribution statement

**Yuanmei Zhang:** Writing – original draft, Investigation, Methodology, Conceptualization. **Yiqi Cao:** Writing – review & editing, Conceptualization. **Bing Chen:** Writing – review & editing, Supervision. **Baiyu Zhang:** Supervision, Writing – review & editing, Conceptualization.

## Declaration of competing interest

The authors declare no competing financial interest.

## References

[1] Geyer R., Jambeck J.R., Law K.L. (2017). Production, use, and fate of all plastics ever made. Sci. Adv..

[2] OECD (2024).

[3] European Bioplastics, Bioplastics market development update 2023, Eur. Bioplastics EV (2023). https://www.european-bioplastics.org/bioplastics-market-development-update-2023-2/. (Accessed 24 September 2024).

[4] Liu L., Xu M., Ye Y., Zhang B. (2022). On the degradation of (micro)plastics: degradation methods, influencing factors, environmental impacts. Sci. Total Environ..

[5] Moyal J., Dave P.H., Wu M., Karimpour S., Brar S.K., Zhong H. (2023). Impacts of biofilm formation on the physicochemical properties and toxicity of microplastics: a concise review. Rev. Environ. Contam. Toxicol..

[6] Peeken I., Primpke S., Beyer B., Gütermann J., Katlein C., Krumpen T. (2018). Arctic sea ice is an important temporal sink and means of transport for microplastic. Nat. Commun..

[7] Van Rossum T. (2021). Marine biodegradability review of plastics. Water Cycle.

[8] Zhi Xiang J.K., Bairoliya S., Cho Z.T., Cao B. (2023). Plastic-microbe interaction in the marine environment: research methods and opportunities. Environ. Int..

[9] Ford H.V., Jones N.H., Davies A.J., Godley B.J., Jambeck J.R., Napper I.E. (2022). The fundamental links between climate change and marine plastic pollution. Sci. Total Environ..

[10] Haque F., Fan C. (2023). Fate of microplastics under the influence of climate change. iScience.

[11] Wei X.-F., Yang W., Hedenqvist M.S. (2024). Plastic pollution amplified by a warming climate. Nat. Commun..

[12] Sudhakar M., Doble M., Murthy P.S., Venkatesan R. (2008). Marine microbe-mediated biodegradation of low- and high-density polyethylenes. Int. Biodeterior. Biodegrad..

[13] Giacomucci L., Raddadi N., Soccio M., Lotti N., Fava F. (2020). Biodegradation of polyvinyl chloride plastic films by enriched anaerobic marine consortia. Mar. Environ. Res..

[14] Khandare S.D., Chaudhary D.R., Bhavanath J. (2021). Marine bacterial biodegradation of low-density polyethylene (LDPE) plastic. Biodegradation.

[15] Law K.L., Thompson R.C. (2014). Microplastics in the seas. Science.

[16] Liu P., Zhan X., Wu X., Li J., Wang H., Gao S. (2020). Effect of weathering on environmental behavior of microplastics: properties, sorption and potential risks. Chemosphere.

[17] Amaral-Zettler L.A., Zettler E.R., Mincer T.J. (2020). Ecology of the plastisphere. Nat. Rev. Microbiol..

[18] Sooriyakumar P., Bolan N., Kumar M., Singh L., Yu Y., Li Y. (2022). Biofilm formation and its implications on the properties and fate of microplastics in aquatic environments: a review. J Hazard Mater Adv.

[19] Du Y., Liu X., Dong X., Yin Z. (2022). A review on marine plastisphere: biodiversity, formation, and role in degradation. Comput. Struct. Biotechnol. J..

[20] Dang H., Lovell C.R. (2016). Microbial surface colonization and biofilm development in marine environments. Microbiol. Mol. Biol. Rev..

[21] Tabraiz S., Petropoulos E., Shamurad B., Quintela-Baluja M., Mohapatra S., Acharya K. (2021). Temperature and immigration effects on quorum sensing in the biofilms of anaerobic membrane bioreactors. J. Environ. Manag..

[22] Römling U., Galperin M.Y., Gomelsky M. (2013). Cyclic di-GMP: the first 25 years of a universal bacterial second messenger. Microbiol. Mol. Biol. Rev..

[23] Lin Y.-T., Wang Y.-C., Xue Y.-M., Tong Z., Jiang G.-Y., Hu X.-R. (2024). Decoding the influence of low temperature on biofilm development: the hidden roles of *c*-di-GMP. Sci. Total Environ..

[24] Oberbeckmann S., Kreikemeyer B., Labrenz M. (2018). Environmental factors support the formation of specific bacterial assemblages on microplastics. Front. Microbiol..

[25] Townsley L., Yildiz F.H. (2015). Temperature affects c-di- GMP signalling and biofilm formation in *Vibrio cholerae*. Environ. Microbiol..

[26] Jeong H.-H., Jeong S.-G., Park A., Jang S.-C., Hong S.G., Lee C.-S. (2014). Effect of temperature on biofilm formation by Antarctic marine bacteria in a microfluidic device. Anal. Biochem..

[27] Bisht K., Moore J.L., Caprioli R.M., Skaar E.P., Wakeman C.A. (2021). Impact of temperature-dependent phage expression on Pseudomonas aeruginosa biofilm formation. Npj Biofilms Microbiomes.

[28] Su X., Yang L., Yang K., Tang Y., Wen T., Wang Y. (2022). Estuarine plastisphere as an overlooked source of N_2_O production. Nat. Commun..

[29] Oberbeckmann S., Löder M.G.J., Labrenz M. (2015). Marine microplastic-associated biofilms – a review. Environ. Chem..

[30] Urbanek A.K., Rymowicz W., Mirończuk A.M. (2018). Degradation of plastics and plastic-degrading bacteria in cold marine habitats. Appl. Microbiol. Biotechnol..

[31] Shah A.A., Hasan F., Hameed A., Ahmed S. (2008). Biological degradation of plastics: a comprehensive review. Biotechnol. Adv..

[32] Misic C., Covazzi Harriague A. (2019). Development of marine biofilm on plastic: ecological features in different seasons, temperatures, and light regimes. Hydrobiologia.

[33] Pinnell L.J., Turner J.W. (2020). Temporal changes in water temperature and salinity drive the formation of a reversible plastic-specific microbial community. FEMS Microbiol. Ecol..

[34] Atanasova N., Stoitsova S., Paunova-Krasteva T., Kambourova M. (2021). Plastic degradation by extremophilic bacteria. Int. J. Mol. Sci..

[35] Denaro R., Aulenta F., Crisafi F., Di Pippo F., Cruz Viggi C., Matturro B. (2020). Marine hydrocarbon-degrading bacteria breakdown poly(ethylene terephthalate) (PET). Sci. Total Environ..

[36] Delacuvellerie A., Cyriaque V., Gobert S., Benali S., Wattiez R. (2019). The plastisphere in marine ecosystem hosts potential specific microbial degraders including Alcanivorax borkumensis as a key player for the low-density polyethylene degradation. J. Hazard Mater..

[37] Cao Y., Zhang B., Cai Q., Zhu Z., Liu B., Dong G. (2022). Responses of Alcanivorax species to marine alkanes and polyhydroxybutyrate plastic pollution: importance of the ocean hydrocarbon cycles. Environ. Pollut..

[38] Lv S., Cui K., Zhao S., Li Y., Liu R., Hu R. (2024). Continuous generation and release of microplastics and nanoplastics from polystyrene by plastic-degrading marine bacteria. J. Hazard Mater..

[39] Zhang Y., Cao Y., Chen B., Dong G., Zhao Y., Zhang B. (2024). Marine biodegradation of plastic films by Alcanivorax under various ambient temperatures: bacterial enrichment, morphology alteration, and release of degradation products. Sci. Total Environ..

[40] Cai Q., Zhang B., Chen B., Zhu Z., Lin W., Cao T. (2014). Screening of biosurfactant producers from petroleum hydrocarbon contaminated sources in cold marine environments. Mar. Pollut. Bull..

[41] Koike H., Miyamoto K., Teramoto M. (2023). *Alcanivorax* bacteria as important polypropylene degraders in mesopelagic environments. Appl. Environ. Microbiol..

[42] LaBauve A.E., Wargo M.J. (2012). Growth and laboratory maintenance of *Pseudomonas aeruginosa*. Curr Protoc Microbiol 0.

[43] Suzuki M., Tachibana Y., Oba K., Takizawa R., Kasuya K. (2018). Microbial degradation of poly(ε-caprolactone) in a coastal environment. Polym. Degrad. Stabil..

[44] Wilkes R.A., Aristilde L. (2017). Degradation and metabolism of synthetic plastics and associated products by *Pseudomonas* sp.: capabilities and challenges. J. Appl. Microbiol..

[45] Liu R., Xu H., Zhao S., Dong C., Li J., Wei G. (2024). Polyethylene terephthalate (PET)-degrading bacteria in the pelagic deep-sea sediments of the Pacific Ocean. Environ. Pollut..

[46] Mouafo Tamnou E.B., Tamsa Arfao A., Nougang M.E., Metsopkeng C.S., Noah Ewoti O.V., Moungang L.M. (2021). Biodegradation of polyethylene by the bacterium *Pseudomonas aeruginosa* in acidic aquatic microcosm and effect of the environmental temperature. Environ. Chall..

[47] Dwicania E., Rinanti A., Fachrul M.F. (2019). Biodegradation of LLDPE plastic by mixed bacteria culture of Pseudomonas aeruginosa and *Brevibacterium* sp. J Phys Conf Ser.

[48] Sheikh H.I., Najiah M., Fadhlina A., Laith A.A., Nor M.M., Jalal K.C.A. (2022). Temperature upshift mostly but not always enhances the growth of vibrio species: a systematic review. Front. Mar. Sci..

[49] Leighton R.E., Correa Vélez K.E., Xiong L., Creech A.G., Amirichetty K.P., Anderson G.K. (2023). Vibrio parahaemolyticus and Vibrio vulnificus in vitro colonization on plastics influenced by temperature and strain variability. Front. Microbiol..

[50] Zhai X., Zhang X.-H., Yu M. (2023). Microbial colonization and degradation of marine microplastics in the plastisphere: a review. Front. Microbiol..

[51] Branchu P., Canette A., Medina Fernandez S., Mounier J., Meylheuc T., Briandet R. (2017). Impact of temperature on Marinobacter hydrocarbonoclasticus SP17 morphology and biofilm structure during growth on alkanes. Microbiology.

[52] Morohoshi T., Ogata K., Okura T., Sato S. (2018). Molecular characterization of the bacterial community in biofilms for degradation of poly(3-hydroxybutyrate-co-3-hydroxyhexanoate) films in seawater. Microb. Environ..

[53] Zhang A., Hou Y., Wang Q., Wang Y. (2022). Characteristics and polyethylene biodegradation function of a novel cold-adapted bacterial laccase from Antarctic sea ice psychrophile *Psychrobacter* sp. NJ228. J. Hazard Mater..

[54] Nogi Y., Horikoshi K. (2011). Extremophiles Handbook.

[55] Poli A., Finore I., Romano I., Gioiello A., Lama L., Nicolaus B. (2017). Microbial diversity in extreme marine habitats and their biomolecules. Microorganisms.

[56] Cao Y., Zhang B., Chen B. (2024). Challenging plastic pollution with hydrocarbonoclastic lineages. Trends Biotechnol..

[57] Brakstad O.G., Lofthus S., Ribicic D., Netzer R., Margesin R. (2017). Psychrophiles: From Biodiversity to Biotechnology.

[58] Gregson B.H., Metodieva G., Metodiev M.V., Golyshin P.N., McKew B.A. (2020). Protein expression in the obligate hydrocarbon-degrading psychrophile *Oleispira antarctica* RB-8 during alkane degradation and cold tolerance. Environ. Microbiol..

[59] Moreno-Ulloa A., Sicairos Diaz V., Tejeda-Mora J.A., Macias Contreras M.I., Castillo F.D., Guerrero A. (2020). Chemical profiling provides insights into the metabolic machinery of hydrocarbon-degrading deep-sea microbes. mSystems.

[60] Rathore D.S., Sheikh M., Singh S.P., Prasad R., Kumar V., Singh J., Upadhyaya C.P. (2021). Recent Developments in Microbial Technologies.

[61] Oliveira J., Almeida P.L., Sobral R.G., Lourenço N.D., Gaudêncio S.P. (2022). Marine-derived actinomycetes: biodegradation of plastics and formation of pha bioplastics—a circular bioeconomy approach. Mar. Drugs.

[62] Xiang W., Liang Y., Hong S., Wang G., You J., Xue Y. (2022). Degradation of long-chain n-alkanes by a novel thermal-tolerant Rhodococcus strain. Arch. Microbiol..

[63] Pátek M., Grulich M., Nešvera J. (2021). Stress response in Rhodococcus strains. Biotechnol. Adv..

[64] Ghanem N.B., Mabrouk M.E.S., Sabry S.A., El-Badan D.E.S. (2005). Degradation of polyesters by a novel marine Nocardiopsis aegyptia sp. Nov.: application of Plackett-Burman experimental design for the improvement of PHB depolymerase activity. J. Gen. Appl. Microbiol..

[65] Suzuki M., Tachibana Y., Takizawa R., Morikawa T., Takeno H., Kasuya K. (2021). A novel poly(3-hydroxybutyrate)-degrading actinobacterium that was isolated from plastisphere formed on marine plastic debris. Polym. Degrad. Stabil..

[66] Jeon Y., Jin H., Kong Y., Cha H.-G., Lee B.W., Yu K. (2023). Poly(3-hydroxybutyrate) degradation by *Bacillus infantis* sp. isolated from soil and identification of *phaZ* and *bdhA* expressing PHB depolymerase. J. Microbiol. Biotechnol..

[67] Cho J.Y., Lee Park S., Lee H.-J., Kim S.H., Suh M.J., Ham S. (2021). Polyhydroxyalkanoates (PHAs) degradation by the newly isolated marine *Bacillus* sp. JY14. Chemosphere.

[68] Kim S.H., Cho J.Y., Cho D.H., Jung H.J., Kim B.C., Bhatia S.K. (2022). Acceleration of polybutylene succinate biodegradation by *Terribacillus* sp. JY49 isolated from a marine environment. Polymers.

[69] Sarmah P., Rout J. (2018). Efficient biodegradation of low-density polyethylene by Cyanobacteria isolated from submerged polyethylene surface in domestic sewage water. Environ. Sci. Pollut. Res..

[70] Rogers K.L., Carreres-Calabuig J.A., Gorokhova E., Posth N.R. (2020). Micro-by-micro interactions: how microorganisms influence the fate of marine microplastics. Limnol Oceanogr Lett.

[71] Tournier V., Duquesne S., Guillamot F., Cramail H., Taton D., Marty A. (2023). Enzymes' power for plastics degradation. Chem. Rev..

[72] Danso D., Chow J., Streit W.R. (2019). Plastics: environmental and biotechnological perspectives on microbial degradation. Appl. Environ. Microbiol..

[73] Danso D., Schmeisser C., Chow J., Zimmermann W., Wei R., Leggewie C. (2018). New insights into the function and global distribution of polyethylene terephthalate (PET)-degrading bacteria and enzymes in marine and terrestrial metagenomes. Appl. Environ. Microbiol..

[74] Stanica-Ezeanu D., Matei D. (2021). Natural depolymerization of waste poly(ethylene terephthalate) by neutral hydrolysis in marine water. Sci. Rep..

[75] Sarkhel R., Sengupta S., Das P., Bhowal A. (2020). Comparative biodegradation study of polymer from plastic bottle waste using novel isolated bacteria and fungi from marine source. J. Polym. Res..

[76] Fecker T., Galaz-Davison P., Engelberger F., Narui Y., Sotomayor M., Parra L.P. (2018). Active site flexibility as a hallmark for efficient PET degradation by I. sakaiensis PETase. Biophys. J..

[77] Son H.F., Cho I.J., Joo S., Seo H., Sagong H.-Y., Choi S.Y. (2019). Rational protein engineering of thermo-stable PETase from *Ideonella sakaiensis* for highly efficient PET degradation. ACS Catal..

[78] Bollinger A., Thies S., Knieps-Grünhagen E., Gertzen C., Kobus S., Höppner A. (2020). A novel polyester hydrolase from the marine bacterium pseudomonas aestusnigri – structural and functional insights. Front. Microbiol..

[79] Marten E., Müller R.-J., Deckwer W.-D. (2003). Studies on the enzymatic hydrolysis of polyesters I. Low molecular mass model esters and aliphatic polyesters. Polym. Degrad. Stabil..

[80] Nakayama A., Yamano N., Kawasaki N. (2019). Biodegradation in seawater of aliphatic polyesters. Polym. Degrad. Stabil..

[81] Jaeger K.-E., Eggert T. (2002). Lipases for biotechnology. Curr. Opin. Biotechnol..

[82] Yoshida K., Teramoto S., Gong J., Kobayashi Y., Ito H. (2024). Enhanced marine biodegradation of polycaprolactone through incorporation of mucus bubble powder from violet sea snail as protein fillers. Polymers.

[83] Shalem A., Yehezkeli O., Fishman A. (2024). Enzymatic degradation of polylactic acid (PLA). Appl. Microbiol. Biotechnol..

[84] Bagheri A.R., Laforsch C., Greiner A., Agarwal S. (2017). Fate of so-called biodegradable polymers in seawater and freshwater. Glob Chall.

[85] Chamas A., Moon H., Zheng J., Qiu Y., Tabassum T., Jang J.H. (2020). Degradation rates of plastics in the environment. ACS Sustain. Chem. Eng..

[86] Guo F., Liu B., Zhao J., Hou Y., Wu J., Hu H. (2024). Temperature-dependent effects of microplastics on sediment bacteriome and metabolome. Chemosphere.

[87] Royer S.-J., Greco F., Kogler M., Deheyn D.D. (2023). Not so biodegradable: polylactic acid and cellulose/plastic blend textiles lack fast biodegradation in marine waters. PLoS One.

[88] Cheung C.K.H., Not C. (2024). Degradation efficiency of biodegradable plastics in subtropical open-air and marine environments: implications for plastic pollution. Sci. Total Environ..

[89] Huang Q., Hiyama M., Kabe T., Kimura S., Iwata T. (2020). Enzymatic self-biodegradation of poly(l-lactic acid) films by embedded heat-treated and immobilized proteinase K. Biomacromolecules.

[90] Tokiwa Y., Calabia B.P. (2006). Biodegradability and biodegradation of poly(lactide). Appl. Microbiol. Biotechnol..

[91] Huang D., Hu Z.-D., Liu T.-Y., Lu B., Zhen Z.-C., Wang G.-X. (2020). Seawater degradation of PLA accelerated by water-soluble PVA. E-Polymers.

[92] He M., Hsu Y.-I., Uyama H. (2024). Superior sequence-controlled poly(l-lactide)-based bioplastic with tunable seawater biodegradation. J. Hazard Mater..

[93] Zhou W., Bergsma S., Colpa D.I., Euverink G.-J.W., Krooneman J. (2023). Polyhydroxyalkanoates (PHAs) synthesis and degradation by microbes and applications towards a circular economy. J. Environ. Manag..

[94] Suzuki M., Tachibana Y., Kasuya K. (2021). Biodegradability of poly(3-hydroxyalkanoate) and poly(ε-caprolactone) via biological carbon cycles in marine environments. Polym. J..

[95] Dilkes-Hoffman L.S., Lant P.A., Laycock B., Pratt S. (2019). The rate of biodegradation of PHA bioplastics in the marine environment: a meta-study. Mar. Pollut. Bull..

[96] Cao Y., Zhang B., Song X., Dong G., Zhang Y., Chen B. (2024). Polyhydroxybutyrate plastics show rapid disintegration and more straightforward biogeochemical impacts than polyethylene under marine biofragmentation. Environ. Sci. Technol..

[97] Urbanek A.K., Mirończuk A.M., García-Martín A., Saborido A., De La Mata I., Arroyo M. (2020). Biochemical properties and biotechnological applications of microbial enzymes involved in the degradation of polyester-type plastics. Biochim Biophys Acta BBA - Proteins Proteomics.

[98] Deroiné M., Le Duigou A., Corre Y.-M., Le Gac P.-Y., Davies P., César G. (2014). Seawater accelerated ageing of poly(3-hydroxybutyrate-co-3-hydroxyvalerate). Polym. Degrad. Stabil..

[99] Kato C., Honma A., Sato S., Okura T., Fukuda R., Nogi Y. (2019). Poly 3-hydroxybutyrate-co-3-hydroxyhexanoate films can be degraded by the deep-sea microbes at high pressure and low temperature conditions. High Press. Res..

[100] Sekiguchi T., Saika A., Nomura K., Watanabe T., Watanabe T., Fujimoto Y. (2011). Biodegradation of aliphatic polyesters soaked in deep seawaters and isolation of poly(ɛ-caprolactone)-degrading bacteria. Polym. Degrad. Stabil..

[101] Gewert B.,M., Plassmann M., MacLeod M. (2015). Pathways for degradation of plastic polymers floating in the marine environment. Environ Sci Process Impacts.

[102] Zhang Y., Pedersen J.N., Eser B.E., Guo Z. (2022). Biodegradation of polyethylene and polystyrene: from microbial deterioration to enzyme discovery. Biotechnol. Adv..

[103] Andrady A.L., Barnes P.W., Bornman J.F., Gouin T., Madronich S., White C.C. (2022). Oxidation and fragmentation of plastics in a changing environment; From UV-radiation to biological degradation. Sci. Total Environ..

[104] Shyam S., Sarma H., Sarma H., Joshi S. (2023). Land Remediation and Management: Bioengineering Strategies.

[105] Hou L., Majumder E.L.-W. (2021). Potential for and distribution of enzymatic biodegradation of polystyrene by environmental microorganisms. Mater Basel Switz.

[106] Gallego S., Vila J., Tauler M., Nieto J.M., Breugelmans P., Springael D. (2014). Community structure and PAH ring-hydroxylating dioxygenase genes of a marine pyrene-degrading microbial consortium. Biodegradation.

[107] Syranidou E., Karkanorachaki K., Amorotti F., Franchini M., Repouskou E., Kaliva M. (2017). Biodegradation of weathered polystyrene films in seawater microcosms. Sci. Rep..

[108] Liu R., Zhao S., Zhang B., Li G., Fu X., Yan P. (2023). Biodegradation of polystyrene (PS) by marine bacteria in mangrove ecosystem. J. Hazard Mater..

[109] Kim Y.-B., Kim S., Park C., Yeom S.-J. (2024). Biodegradation of polystyrene and systems biology-based approaches to the development of new biocatalysts for plastic degradation. Curr. Opin. Syst. Biol..

[110] Zhang Y., Zheng X., Kong D., Yan H., Liu Z. (2021). Enhanced North Pacific subtropical gyre circulation during the late Holocene. Nat. Commun..

[111] Kaushal J., Khatri M., Arya S.K. (2021). Recent insight into enzymatic degradation of plastics prevalent in the environment: a mini - review. Clean Eng. Technol..

[112] Sohail M., Siddiqi R., Ahmad A., Khan S.A. (2009). Cellulase production from Aspergillus niger MS82: effect of temperature and pH. N. Biotech..

[113] Lin C., Zhang H. (2025). Polymer biodegradation in aquatic environments: a machine learning model informed by meta-analysis of structure-biodegradation relationships. Environ. Sci. Technol..

[114] Sung C.-C., Tachibana Y., Suzuki M., Hsieh W.-C., Kasuya K. (2016). Identification of a poly(3-hydroxybutyrate)-degrading bacterium isolated from coastal seawater in Japan as Shewanella sp. Polym. Degrad. Stabil..

[115] Wang S., Lydon K.A., White E.M., Grubbs J.B., Lipp E.K., Locklin J. (2018). Biodegradation of poly(3-hydroxybutyrate- *co* -3-hydroxyhexanoate) plastic under anaerobic sludge and aerobic seawater conditions: gas evolution and microbial diversity. Environ. Sci. Technol..

[116] Tachibana Y., Kageyama K., Suzuki M., Koshigumo H., Takeno H., Tachibana Y. (2019). Microbial composition and polymer hydrolytic activity of Japanese washed-rind cheeses. Polym. Degrad. Stabil..

[117] Saito Y., Honda M., Yamashita T., Furuno Y., Kato D., Abe H. (2023). Marine bacterial enzyme degrades polyamide 4 into gamma-aminobutyric acid oligomers. Polym. Degrad. Stabil..

[118] Wu B., Wu H., Xu S.-M., Wang Y.-Z. (2023). Comparative study of the aging degradation behaviors of PET under artificially accelerated and typical marine environment. Polym. Degrad. Stabil..

[119] Dimassi S.N., Hahladakis J.N., Chamkha M., Ahmad M.I., Al-Ghouti M.A., Sayadi S. (2024). Investigation on the effect of several parameters involved in the biodegradation of polyethylene (PE) and low-density polyethylene (LDPE) under various seawater environments. Sci. Total Environ..

[120] Shin M., Kim H., Kim S., Kim H.J., Oh D.X., Park J. (2024). Biodegradation behavior of polyesters with various internal chemical structures and external environmental factors in real seawater. Polym. Test..

[121] Le Gué L., Davies P., Arhant M., Vincent B., Tanguy E. (2023). Mitigating plastic pollution at sea: natural seawater degradation of a sustainable PBS/PBAT marine rope. Mar. Pollut. Bull..

[122] Lott C., Eich A., Makarow D., Unger B., Van Eekert M., Schuman E. (2021). Half-life of biodegradable plastics in the marine environment depends on material, habitat, and climate zone. Front. Mar. Sci..

[123] Lee J., Kim S., Park S.B., Shin M., Kim S., Kim M.-S. (2024). Mimicking real-field degradation of biodegradable plastics in soil and marine environments: from product utility to end-of-life analysis. Polym. Test..

[124] Gao R., Sun C. (2021). A marine bacterial community capable of degrading poly(ethylene terephthalate) and polyethylene. J. Hazard Mater..

[125] Eich A., Mildenberger T., Laforsch C., Weber M. (2015). Biofilm and diatom succession on polyethylene (PE) and biodegradable plastic bags in two marine habitats: early signs of degradation in the pelagic and benthic zone?. PLoS One.

